# Effect of Amino Acids on the Generation of Ginsenoside Rg3 Epimers by Heat Processing and the Anticancer Activities of Epimers in A2780 Human Ovarian Cancer Cells

**DOI:** 10.1155/2016/3146402

**Published:** 2016-03-09

**Authors:** Jun Yeon Park, Pilju Choi, Dahae Lee, Taejung Kim, Eun Bee Jung, Buyng-Su Hwang, Ki Sung Kang, Jungyeob Ham

**Affiliations:** ^1^College of Korean Medicine, Gachon University, Seongnam 461-701, Republic of Korea; ^2^KIST Gangneung Institute of Natural Products, Korea Institute of Science and Technology, Gangneung 210-340, Republic of Korea

## Abstract

Ginsenosides are the active components of* Panax ginseng*. Many research studies indicate that these deglycosylated, less-polar ginsenosides have better bioactivity than the major ginsenosides. In the present study, we sought to verify the enhanced anticancer effect of* P. ginseng* extract after undergoing the Maillard reaction as well as elucidate the underlying mechanism of action. The effects of 9 amino acids were tested; among them, the content of 20(S)-Rg3 in the ginseng extract increased to more than 30, 20, and 20% when processed with valine, arginine, and alanine, respectively, compared with that after normal heat processing. The ginseng extract that was heat-processed with arginine exhibited the most potent inhibitory effect on A2780 ovarian cancer cell proliferation. Therefore, the generation of 20(S)-Rg3 was suggested to be involved in this effect. Moreover, the inhibitory effect of 20(S)-Rg3 on A2780 cell proliferation was significantly stronger than that of 20(R)-Rg3. Protein expression levels of cleaved caspase-3, caspase-8, caspase-9, and PARP in the A2780 ovarian cancer cells markedly increased, whereas the expression of BID decreased after 20(S)-Rg3 treatment. Therefore, we confirmed that the anticancer effects of the products of ginseng that was heat-processed with arginine are mediated mainly via the generation of the less-polar ginsenoside 20(S)-Rg3.

## 1. Introduction

The use of naturally occurring compounds for the design of novel anticancer drugs has increased in recent years, especially owing to increasing demand for natural remedies among cancer patients [1–4]. Experimental as well as clinical studies have shown that ginseng and its components are beneficial for chemotherapy since they inhibit proliferation and induce apoptosis in various malignancies, including ovarian cancer [5–8].

**Figure 1:**
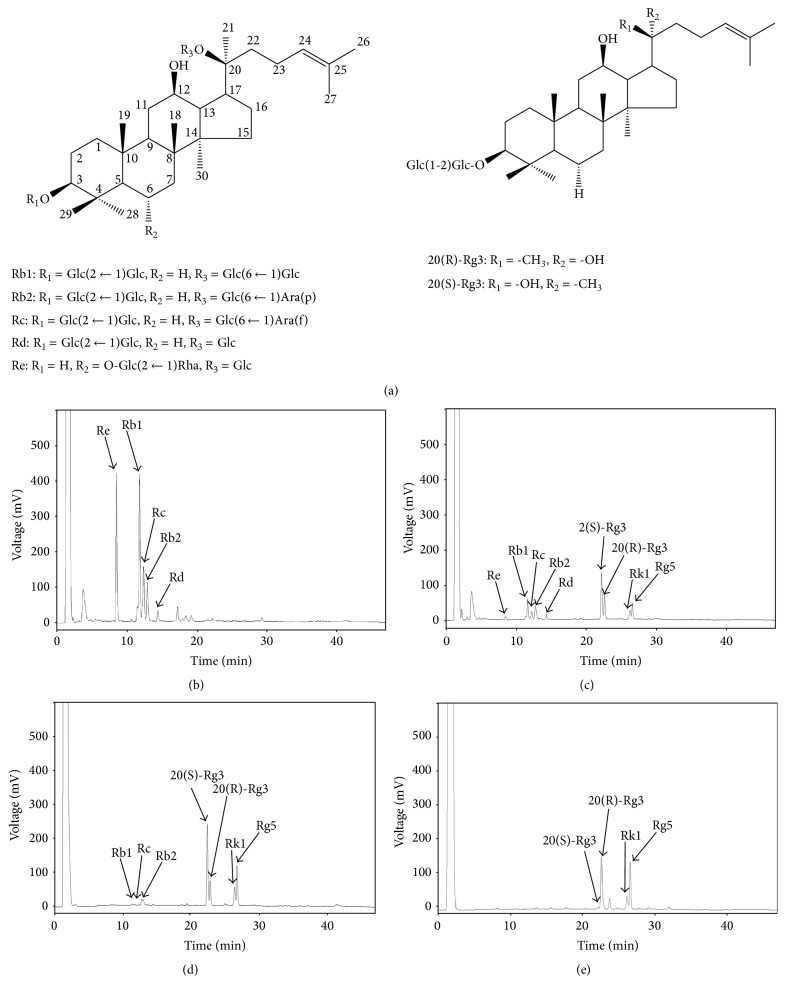
Changes in the concentrations of ginsenosides in* Panax ginseng* extract after heat processing with amino acids. (a) Structures of major and deglycosylated ginsenosides. (b) HPL chromatogram of* Panax ginseng* extract before heat processing. (c) HPL chromatogram of* Panax ginseng* extract after heat processing. (d) HPL chromatogram of* Panax ginseng* extract after heat processing with arginine. (e) HPL chromatogram of* Panax ginseng* extract after heat processing with leucine. -Glc: D-glucopyranosyl, -Rha: L-rhamnopyranosyl, -Ara(f): L-arabinofuranosyl, and -Ara(p): L-arabinopyranosyl.

The roots of ginseng contain dammarane-based saponins, which are composed of 1 to 4 glycosyl moieties combined with a dammarane backbone. The saponins that are present in high concentrations in ginseng are the major ginsenosides Rb1, Rb2, Rc, Rd, and Re (Figure 1) [9, 10]. In addition, less-polar ginsenosides such as Rg3 and Rg5 can be obtained from the deglycosylation of the major ginsenosides [11]. Many research studies indicate that these deglycosylated, less-polar ginsenosides have better bioactivity than the major ginsenosides [5, 12, 13]. Ginsenoside Rg3 activated caspase-3 and Bax protein in human hepatocellular carcinoma cells [14]. Ginsenoside Rg3 also suppressed lung cancer migration and invasion by inhibiting the transforming growth factor-beta 1-induced epithelial-mesenchymal transition [15]. Ginsenoside Rg5 blocked cell cycle of hepatocellular carcinoma cells at the Gl/S transition phase by downregulating cyclin E-dependent kinase activity [16]. Ginsenoside Rk1 induces apoptosis, G1 phase arrest, and autophagy in SK-HEP-1 cells [17, 18]. Therefore, the development of ginseng extract containing an increased concentration of deglycosylated ginsenosides has been investigated.

Heat processing and enzyme-based methods are two major techniques that can achieve deglycosylation of ginsenosides [9]. The Maillard reaction of amino acids with sugars is a nonenzymatic browning reaction that takes place during the processing, cooking, and storage of foods. It is well known that Maillard reaction products produced in red ginseng have antioxidant activity [19, 20]. We have extensively studied and developed novel heat processing methods that facilitate the deglycosylation of major ginsenosides Rb1, Rb2, Rc, Rd, and Re [4, 10]. Recently, we identified the importance of the Maillard reaction in the generation of less-polar ginsenosides with a protective effect in the kidney [21]. In the present study, we sought to further improve the anticancer effect of* Panax ginseng* extract via the Maillard reaction as well as elucidate its mechanism of action.

## 2. Materials and Methods

### 2.1. Chemicals and Reagents

Ginsenoside standards Rb1, Rb2, Rc, Rd, Re, 20(S)-Rg3, 20(R)-Rg3, Rk1, and Rg5 were purchased from Ambo Institute (Seoul, Korea). L-Amino acid standards alanine, arginine, glutamine, glycine, leucine, lysine, serine, trans-4-hydroxy-L-proline, and valine were purchased from Sigma Aldrich (Saint Louis, MO, USA). Monoclonal antibodies against cleaved caspase-8 and *β*-actin and polyclonal antibodies against cleaved caspase-3, cleaved caspase-9, BH3-interacting domain death (BID) agonist, and poly (ADP-ribose) polymerase (PARP) were purchased from Cell Signaling Technology, Inc. (Danvers, MA, USA).

### 2.2. Maillard Reaction Model Experiment Using Ginseng Extract with Amino Acids

Four-year-old ginseng (*P. ginseng*) was purchased from a local ginseng market in Seoul (Korea). Dried ginseng was ground until it could pass through an 80-mesh sieve. It was then extracted under reflux with 50% EtOH three times at 70°C for 2 h and filtered through filter paper (Advantec, Tokyo, Japan). The solvent was evaporated in vacuo to give a 50% EtOH extract with a yield of about 20%, by weight, of the original ginseng powder. The heat processing conditions were designed such that the total ginsenosides contained in the ginseng extract reacted with the same molar concentrations of amino acids. The mixtures were steamed together at 120°C for 3 h as reported previously [22]. After drying at 50°C for 3 days, the untreated and heat-processed ginseng extracts with amino acids were prepared.

### 2.3. Antiproliferative Effect on A2780 Ovarian Cancer Cells

The human ovarian cancer A2780 cell line was purchased from the American Type Culture Collection (ATCC, Manassas, VA). The cells were grown in RPMI1640 medium (Cellgro, Manassas, VA) supplemented with 10% fetal bovine serum (Gibco BRL, Carlsbad, MD), 100 units/mL penicillin, and 100 *μ*g/mL streptomycin. They were incubated at 37°C in a humidified atmosphere with 5% CO_2_. The Cell Counting Kit-8 (CCK-8, Dojindo Laboratories, Japan) was used to determine cell proliferation according to the manufacturer's recommendations. Briefly, cells were seeded in 96-well plates at a density of 1 × 10^4^ cells/well and incubated for 24 h at 37°C. The cells were treated with different concentrations of compounds. After incubation for 24 h, 10 *μ*L of the kit reagent was added to each well, and the cells were incubated for an additional hour. Cell proliferation was measured by scanning with a microplate reader at 450 nm. Control cells were exposed to culture media containing 0.5% v/v dimethyl sulfoxide (DMSO).

### 2.4. Analysis and Structural Confirmation of Ginsenosides

The analytical reversed-phase high-performance liquid chromatography (HPLC) system comprised a solvent degasser (Agilent, G1322A), binary pump (Agilent, G1312C), an autosampler (Agilent, G1329B), and model ZAM 3000 Evaporative Light Scattering Detector (ELSD) (Young Lin, South Korea). ELSD conditions were optimized in order to achieve maximum sensitivity; the temperature of the nebulizer was set for 50°C, and N_2_ was used as the nebulizing gas at a pressure of 2.0 bar. A Phenomenex Luna C18 column (150 × 4.6 mm, 5 *μ*m) was used, and the mobile phase comprised binary gradient of solvent A (acetonitrile : water : 5% acetic acid in water = 15 : 80 : 5) and solvent B (acetonitrile : water = 80 : 20) at a flow rate of 1.0 mL/min. The gradient flow program was as follows: initial 0% B; 6 min, 30% B; 18 min, 50% B; 30 min, 100% B; 37 min, 100% B; 42 min, 0% B.

### 2.5. Flow Cytometric Assay

To detect changes in cell cycle distribution, the treated cells were collected, washed with cold PBS, and fixed in 70% ethanol at 4°C for 30 min. The cells were then washed twice with PBS and resuspended in 500 *μ*L of a propidium iodide (PI) staining solution containing 40 *μ*g/mL PI and 20 *μ*g/mL RNase A in PBS. Then, the cells were incubated at room temperature for 30 min in the dark and analyzed with a FACSCalibur flow cytometer (Becton-Dickinson, San Jose, CA, USA) and the ModFit LT version 2.0 computer program.

### 2.6. Western Blotting Analysis

Cells (8 × 10^5^ cells) grown in 60 mm dishes were treated with the indicated concentration of samples for 24 h. Whole-cell extracts were then prepared according to the manufacturer's instructions using RIPA buffer (Cell Signaling, MA, USA) supplemented with 1x protease inhibitor cocktail and 1 mM phenylmethylsulfonyl fluoride (PMSF) [23]. Proteins (whole-cell extracts, 30 *μ*g/lane) were separated by electrophoresis in a precast 4–15% Mini-PROTEAN TGX gel (Bio-Rad, CA, USA), blotted onto PVDF transfer membranes, and analyzed with epitope-specific primary and secondary antibodies. Bound antibodies were visualized using ECL Advance Western Blotting Detection Reagents (GE Healthcare, UK) and a LAS 4000 imaging system (Fujifilm, Japan).

### 2.7. Statistical Analysis

Statistical significance was determined through analysis of variance (ANOVA) followed by a multiple comparison test with a Bonferroni adjustment. *P* values of less than 0.05 were considered statistically significant.

## 3. Results and Discussion

In the present study, we developed a novel method of converting ginsenosides, the major active components of ginseng, and enhancing the anticancer effect of ginseng by high-temperature amino acid processing.

*P. ginseng* contains a high concentration of ginsenosides.  Figure 1(a) shows the structure of the major ginsenosides contained in ginseng, including Rb1, Rb2, Rc, Rd, Rg3, and Re. The typical ginsenosides in raw ginseng include Re, Rb1, Rc, Rb2, and Rd (Figure 1(b)). As shown in Figure 1(c), when Rb1, Rb2, Rc, Rd, Rg1, and Re are heat-processed, glucose, which is a glycoside located at position 20, may be dissociated and subsequent dehydration may occur at position 20, such that ginsenosides Rb1, Rb2, Rc, Rd, Rg1, and Re are converted into Rg3, Rg5, and Rk1 (compare Figures 1(b) and 1(c)). In the present study, we further examined the effects of amino acids on the generation of these less-polar ginsenosides. The effects of 9 amino acids (alanine, arginine, glutamine, glycine, leucine, lysine, serine, trans-4-hydroxy-L-proline, and valine) were tested; among them, 5 amino acids (glutamine, leucine, valine, arginine, and alanine) had a significant influence on the generation of less-polar ginsenosides during heat processing. The concentration of 20(R)-Rg3 in the ginseng extract increased to more than 50 and 100% when processed with glutamine and leucine, respectively, compared to 20(R)-Rg3 concentration after normal heat processing (Table 1). In addition, the contents of the ginsenoside 20(S)-Rg3 decreased in the ginseng extract, while those of ginsenosides Rk1 and Rg5 increased when the extract was heat-processed with glutamine or leucine. Interestingly, the concentration of 20(S)-Rg3 in the ginseng extract increased to more than 30, 20, and 20% when it is processed with valine, arginine, and alanine, respectively, compared to that obtained after normal heat processing (Table 1). In addition, the concentration of ginsenoside 20(R)-Rg3 decreased, while those of ginsenosides Rk1 and Rg5 increased in the extract when it was heat-processed with valine, arginine, and alanine. Therefore, certain amino acids were found to affect the generation of less-polar ginsenosides. Among them, two representative amino acids, leucine and arginine, were selected for the evaluation of anticancer effects. Figures 1(d) and 1(e) show the representative HPL chromatogram of the* Panax ginseng* extract after heat processing with arginine and leucine. These heat processing conditions were selected because they generated the greatest total amount of less-polar ginsenosides and were stereospecific in their generation of Rg3 epimers (Table 1, Figure 1).

**Figure 2:**
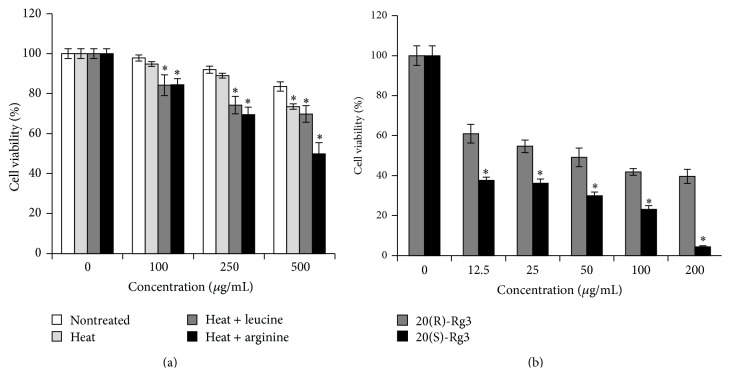
Changes in the effects of* Panax ginseng* extract on A2780 cell proliferation after heat processing with or without amino acids. (a) Cells were treated with nontreated or heat-processed* Panax ginseng* extract with or without amino acids at different concentrations (100, 250, and 500 *μ*g/mL) for 24 h. (b) Cells were treated with ginsenoside 20(S)-Rg3 or ginsenoside 20(R)-Rg3 at different concentrations (12.5, 25, 50, 100, and 200 *μ*g/mL) for 24 h. Relative cell proliferation was measured by the CCK-8 assay. Each value represents the mean ± SD of three independent experiments. ^*∗*^ *P* < 0.05 compared with nontreated value.

The percent viability of A2780 ovarian cancer cells after treatment with ginseng extract (with leucine or arginine) and compounds (20(R)-Rg3 and 20(S)-Rg3) is shown in Figure 2. In Figure 2(a), each ginseng extract inhibited A2780 cell proliferation in a dose-dependent manner. Among them, the ginseng extract that was heat-processed with arginine exhibited the most potent inhibition. Therefore, the generation of 20(S)-Rg3 was suggested to be involved in the increased anticancer effect. The effect of 20(R)-Rg3 and 20(S)-Rg3 on the viability of A2780 is shown in Figure 2(b). It can be seen that 20(R)-Rg3 and 20(S)-Rg3 inhibited A2780 cell proliferation in a dose-dependent manner, and the inhibitory effect of 20(S)-Rg3 was significantly stronger than that of 20(R)-Rg3. The effect of 20(S)-Rg3 on the cell cycle distribution at 24 h was tested using flow cytometric assay. However, treatment with 20(S)-Rg3 did not affect A2780 cancer cell cycle arrest (data not shown). Therefore, it might be reasonable to identify apoptosis as a mechanistic pathway of heat-processed ginseng extract with arginine or 20(S)-Rg3.

Dysregulation of apoptosis leads to various human pathologies including cancer [24, 25]. Intrinsic apoptosis is induced through the activation of specific signaling pathways, while extrinsic apoptosis is initiated through transmembrane death receptors [26, 27]. Initiation and execution of these processes are regulated by initiating factors, including proteases such as caspases and the Bcl-2 family of proteins [28]. Caspase-8 may in turn either directly cleave effector caspases, such as caspase-3, or amplify the death signal through the translocation of Bcl-2 family member BID to the mitochondria to activate the downstream components of the mitochondria apoptotic pathway [29, 30]. The cleavage of PARP is also an important indicator of caspase-3 activation during apoptosis [31].

**Figure 3:**
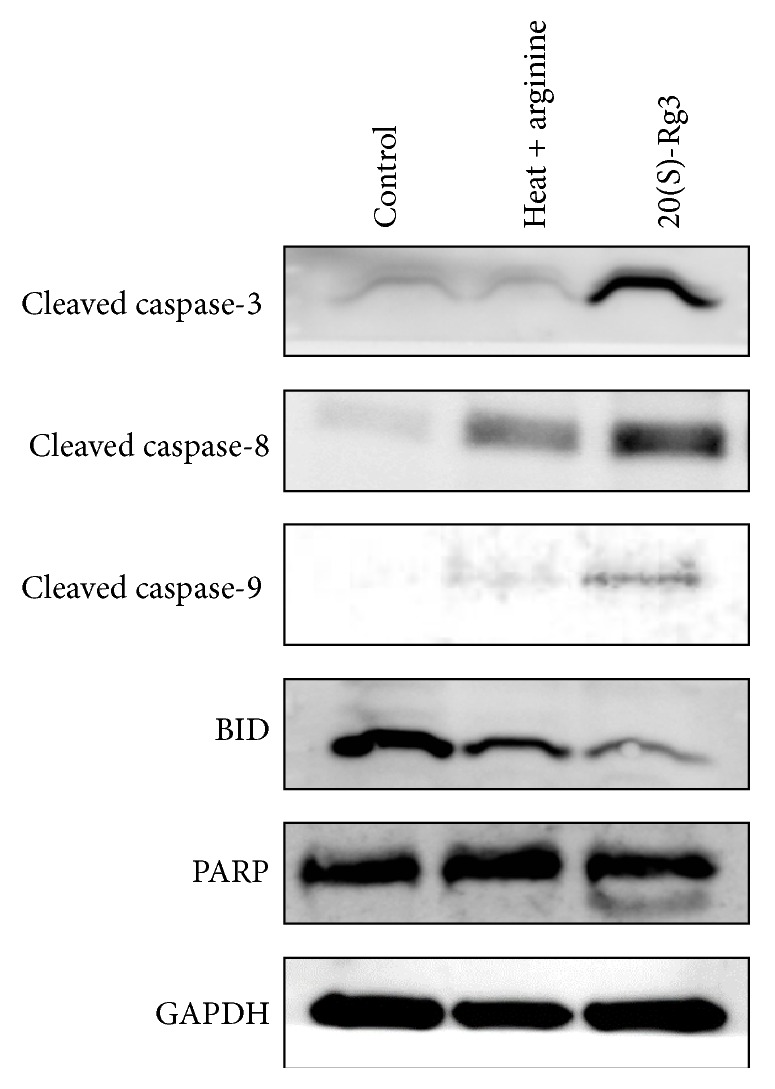
Effects of* Panax ginseng* extract heat-processed with arginine and ginsenoside 20(S)-Rg3 on apoptosis in A2780 cells. Western blotting results showing the levels of cleaved caspase-3, cleaved caspase-8, cleaved caspase-9, BID, and PARP in A2780 cells treated with heat-processed* Panax ginseng* extract with arginine (500 *μ*g/mL) and ginsenoside 20(S)-Rg3 (12.5 *μ*g/mL) for 24 h. Thirty micrograms of each protein was separated by SDS-PAGE. GAPDH was used as an internal control.

Figure 3 shows the protein expressions of cleaved caspase-3, cleaved caspase-8, cleaved caspase-9, BID, and PARP in the control and experimental groups (treatment with arginine and 20(S)-Rg3). Protein expression levels of cleaved caspase-3, caspase-8, and caspase-9 in A2780 ovarian cancer cells markedly increased after 20(S)-Rg3 treatment. In addition, a decrease in protein expression of BID and an increase in cleavage of PARP were noted after 20(S)-Rg3 treatment. Therefore, the apoptotic effects of the products of ginseng that was heat-processed with arginine were confirmed to be mainly mediated by the generation of the less-polar ginsenoside 20(S)-Rg3.

In conclusion, the modulation of the anticancer effect of a* P. ginseng* extract via Maillard reaction was verified and its mechanism of action was further outlined. The heat-processed ginseng had abundance of ginsenosides Rg3, Rg5, and Rk1. After treatment with amino acids, the ginseng had a higher concentration of 20(S)-Rg3 and 20(R)-Rg3 and thus had an increased medicinal effect. We demonstrated that 20(S)-Rg3 suppressed the growth of A2780 ovarian cancer cells in vitro. Therefore, heat processing by Maillard reaction is a useful method to enhance the anticancer effect of ginseng by increasing the content of 20(S)-Rg3.

## Figures and Tables

**Table 1:** Changes in contents of ginsenosides (*μ*g/mg).

Ginsenosides	Re	Rb1	Rc	Rb2	Rd	20(S)-Rg3	20(R)-Rg3	Rk1	Rg5
Nontreated	26.1	35.0	15.1	19.7	4.1	—	—	—	—
Heat	2.0	1.4	7.8	3.4	12.1	31.6	20.5	7.3	13.9
Heat/glutamine	—	—	—	—	—	7.4	30.4	14.1	48.1
Heat/leucine	—	—	—	—	—	1.8	42.7	11.7	35.1
Heat/valine	4.5	5.1	2.0	8.9	1.0	41.8	10.3	8.5	18.0
Heat/arginine	—	10.6	0.4	7.7	—	38.5	11.0	10.4	21.3
Heat/alanine	3.7	5.5	2.3	9.4	—	39.0	12.7	8.2	18.0
